# Supplementary material for: Auditable cross-instrument detection of unusual multivariate psychiatric response configurations using a semantically aligned covariance subspace
Source: medRxiv. 2026 Jun 12:2026.05.22.26353902. Preprint. [Version 2] doi: 10.64898/2026.05.22.26353902 (PMC13278192; doi:10.64898/2026.05.22.26353902)
Supplement: Supplementary file 1 [file NIHPP2026.05.22.26353902v2-supplement-3.pdf]

medRxiv preprint doi: https://doi.org/10.64898/2026.05.22.26353902; this version posted June 12, 2026. The copyright holder for this preprint (which was not certified by peer review) is the author/funder, who has granted medRxiv a license to display the preprint in perpetuity. This article is a US Government work. It is not subject to copyright under 17 USC 105 and is also made available for use under a CC0 license.

Supplementary Appendix 1. Structured analytic summaries of retained multivariate outliers

Scope and interpretive constraints

The case summaries below are analytic descriptions of response configurations in the finalized semantically aligned subspace. They are not clinical case formulations, diagnostic judgments, or treatment recommendations. Cases were retained only if they exceeded the cohort-specific empirical Mahalanobis threshold after Ledoit-Wolf covariance regularization *and* did not endorse any item at the Likert ceiling on any instrument. Age and sex were regressed out before subspace scoring. Respondent study identifiers have been removed in accordance with data use requirements. Cases are identified by their anonymized case labels only.

Because several questionnaire items were reverse-coded during preprocessing, interpretation is based on the joint configuration of items and component structure, rather than on any single coded response in isolation. For clarity, item labels are reported in their original questionnaire wording.

Older-adult cohort: HRS

Cohort size: 4,413
Retained subspace dimensionality: 10
Retained cross-instrument outliers: 7

Case HRS-1

Mahalanobis distance: 5.2901
Empirical rank: 84/4,413 (top 1.903%)
Non-zero items: 22/27

Dominant component	Z-score	Highest-loading items contributing to separation
C5	-3.202	"I felt that everything I did was an effort."; "I felt hands trembling."; "I enjoyed life."; "I was happy."; "In the last month, how often have you been upset because of something that happened unexpectedly?"
C3	-1.819	"I had a fear of the worst happening."; "I felt lonely."; "I had a fear of dying."; "I felt sad."; "I felt depressed."
C9	-1.687	"How often do you feel really rested when you wake up in the morning?"; "In the last month, how often have you felt that things were going your way?"; "In the last month, how often have you found that you could not cope with all the things that you had to do?"; "How often do you have trouble falling asleep?"; "In the last month, how often have you felt nervous and stressed?"

medRxiv preprint doi: https://doi.org/10.64898/2026.05.22.26353902; this version posted June 12, 2026. The copyright holder for this preprint (which was not certified by peer review) is the author/funder, who has granted medRxiv a license to display the preprint in perpetuity. This article is a US Government work. It is not subject to copyright under 17 USC 105 and is also made available for use under a CC0 license.

Analytic summary.

This respondent's outlying position was driven by nonzero responses on upset-over-unexpected-events, fear-of-the-worst-happening, fear-of-dying, hands-trembling, trouble-falling-asleep, and nervous/stressed items, together with zero responses on loneliness-, sadness-, depression-, and effort-labeled items. The positive-valence items "I enjoyed life" and "I was happy," as well as restfulness and coping-related items, also contributed to the separation. The unusual feature is the co-occurrence of these present and absent items within the same response pattern.

Case HRS-2

Mahalanobis distance: 5.1196
Empirical rank: 128/4,413 (top 2.901%)
Non-zero items: 21/27

Dominant component	Z-score	Highest-loading items contributing to separation
C2	+2.690	"I enjoyed life."; "I was happy."; "How often do you feel really rested when you wake up in the morning?"; "I felt that everything I did was an effort."; "In the last month, how often have you felt that you were unable to control the important things in your life?"
C9	−2.220	"How often do you feel really rested when you wake up in the morning?"; "In the last month, how often have you felt that things were going your way?"; "In the last month, how often have you found that you could not cope with all the things that you had to do?"; "How often do you have trouble falling asleep?"; "In the last month, how often have you felt nervous and stressed?"
C5	−2.195	"I felt that everything I did was an effort."; "I felt hands trembling."; "I enjoyed life."; "I was happy."; "In the last month, how often have you been upset because of something that happened unexpectedly?"

Analytic summary.

This respondent's outlying position was driven by nonzero responses on the positive-valence items "I enjoyed life" and "I was happy," restfulness-on-waking, trouble-falling-asleep, things-going-your-way, upset-over-unexpected-events, and could-not-cope items, together with zero responses on the effort item. Hands-trembling also contributed at lower level. The unusual feature is the joint placement of positive-valence, sleep-related, and stress-related items together with a zero response on the effort item.

Case HRS-3

Mahalanobis distance: 5.2662
Empirical rank: 95/4,413 (top 2.153%)
Non-zero items: 22/27

Analytic summary.

This respondent's outlying position was driven by nonzero responses on fear-of-the-worst-happening, fear-of-dying, loneliness-, sadness-, and depression-labeled items, upset-over-unexpected-events, trouble-falling-asleep, and control-related items, together with zero responses on "I enjoyed life," "I was happy," and the effort item. Restfulness-on-waking also contributed. The unusual feature is the co-occurrence of nonzero responses on several fear-, sadness-, and control-related items with zero responses on the positive-valence and effort items.

Case HRS-4

Mahalanobis distance: 4.9443
Empirical rank: 191/4,413 (top 4.328%)

medRxiv preprint doi: https://doi.org/10.64898/2026.05.22.26353902; this version posted June 12, 2026. The copyright holder for this preprint (which was not certified by peer review) is the author/funder, who has granted medRxiv a license to display the preprint in perpetuity. This article is a US Government work. It is not subject to copyright under 17 USC 105 and is also made available for use under a CC0 license.

Dominant component	Z-score	Highest-loading items contributing to separation
C2	-2.915	"I enjoyed life."; "I was happy."; "How often do you feel really rested when you wake up in the morning?"; "I felt that everything I did was an effort."; "In the last month, how often have you felt that you were unable to control the important things in your life?"
C3	$+2.478$	"I had a fear of the worst happening."; "I felt lonely."; "I had a fear of dying."; "I felt sad."; "I felt depressed."
C6	$+1.948$	"In the last month, how often have you been upset because of something that happened unexpectedly?"; "In the last month, how often have you felt confident about your ability to handle your personal problems?"; "I had a fear of dying."; "How often do you have trouble falling asleep?"; "In the last month, how often have you been able to control irritations in your life?"

Non-zero items: 22/27

Dominant component	Z-score	Highest-loading items contributing to separation
C2	-1.847	"I enjoyed life."; "I was happy."; "How often do you feel really rested when you wake up in the morning?"; "I felt that everything I did was an effort."; "In the last month, how often have you felt that you were unable to control the important things in your life?"
C3	$+1.619$	"I had a fear of the worst happening."; "I felt lonely."; "I had a fear of dying."; "I felt sad."; "I felt depressed."
C0	$+1.607$	"I felt sad."; "I felt lonely."; "I felt faint."; "I was happy."; "I felt depressed."

Analytic summary.

This respondent's outlying position was driven by nonzero responses on fear-of-the-worst-happening, loneliness-, depression-, and faintness-labeled items, together with zero responses on "I enjoyed life," "I was happy," the effort item, and the sadness item in one dominant component. Restfulness-on-waking and inability-to-control-important-things also contributed. The unusual feature is the joint placement of faintness-, fear-, and selected mood-labeled items with zero responses on positive-valence and effort items.

Case HRS-5

Mahalanobis distance: 5.1654
Empirical rank: 120/4,413 (top 2.719%)
Non-zero items: 22/27

Dominant component	Z-score	Highest-loading items contributing to separation
C2	-2.903	"I enjoyed life."; "I was happy."; "How often do you feel really rested when you wake up in the morning?"; "I felt that everything I did was an effort."; "In the last month, how often have you felt that you were unable to control the important things in your life?"
C3	$+2.893$	"I had a fear of the worst happening."; "I felt lonely."; "I had a fear of dying."; "I felt sad."; "I felt depressed."
C6	$+2.581$	"In the last month, how often have you been upset because of something that happened unexpectedly?"; "In the last month, how often have you felt confident about your ability to handle your personal problems?"; "I had a fear of dying."; "How often do you have trouble falling asleep?"; "In the last month, how often have you been able to control irritations in your life?"

medRxiv preprint doi: https://doi.org/10.64898/2026.05.22.26353902; this version posted June 12, 2026. The copyright holder for this preprint (which was not certified by peer review) is the author/funder, who has granted medRxiv a license to display the preprint in perpetuity. This article is a US Government work. It is not subject to copyright under 17 USC 105 and is also made available for use under a CC0 license.

Analytic summary.

This respondent's outlying position was driven by nonzero responses on loneliness-, sadness-, and depression-labeled items, fear-of-dying, upset-over-unexpected-events, confidence/control-related items, and trouble-falling-asleep, together with zero responses on "I enjoyed life," "I was happy," and the effort item. Restfulness-on-waking also contributed. The unusual feature is the co-occurrence of nonzero responses on several sadness-, fear-, and control-related items with zero responses on the positive-valence and effort items.

Case HRS-6

Mahalanobis distance: 5.0286

Empirical rank: 156/4,413 (top 3.535%)

Non-zero items: 22/27

Dominant component	Z-score	Highest-loading items contributing to separation
C6	−3.325	"In the last month, how often have you been upset because of something that happened unexpectedly?"; "In the last month, how often have you felt confident about your ability to handle your personal problems?"; "I had a fear of dying."; "How often do you have trouble falling asleep?"; "In the last month, how often have you been able to control irritations in your life?"
C4	+2.877	"I could not get going."; "In the last month, how often have you found that you could not cope with all the things that you had to do?"; "In the last month, how often have you felt nervous and stressed?"; "I had a fear of the worst happening."; "In the last month, how often have you felt difficulties were piling up so high that you could not overcome them?"
C3	−2.107	"I had a fear of the worst happening."; "I felt lonely."; "I had a fear of dying."; "I felt sad."; "I felt depressed."

Analytic summary.

This respondent's outlying position was driven by nonzero responses on upset-over-unexpected-events, inability-to-cope, difficulties-piling-up, nervous/stressed, "I could not get going," and fear-related items, together with zero responses on loneliness-, sadness-, and depression-labeled items. Trouble-falling-asleep and control-related items also contributed. The unusual feature is the co-occurrence of nonzero responses on several stress-, coping-, and fear-related items with zero responses on several mood-labeled items.

Case HRS-7

Mahalanobis distance: 5.2535

Empirical rank: 99/4,413 (top 2.243%)

Non-zero items: 22/27

Analytic summary.

This respondent's outlying position was driven by nonzero responses on hands-trembling, upset-over-unexpected-events, early-waking, night-waking, trouble-falling-asleep, restless-sleep, and restfulness-on-waking items, together with zero responses on "I could not get going" and the effort item in two dominant components. The positive-valence items "I enjoyed life" and "I was happy" also contributed. The unusual feature is the joint placement of trembling-, stress-, positive-valence-, and multiple sleep-related items together with zero responses on selected activation-related items.

Younger-adult cohort: Xinxiang

Cohort size: 24,292

Retained subspace dimensionality: 16

Retained cross-instrument outliers: 7

medRxiv preprint doi: https://doi.org/10.64898/2026.05.22.26353902; this version posted June 12, 2026. The copyright holder for this preprint (which was not certified by peer review) is the author/funder, who has granted medRxiv a license to display the preprint in perpetuity. This article is a US Government work. It is not subject to copyright under 17 USC 105 and is also made available for use under a CC0 license.

Dominant component	Z-score	Highest-loading items contributing to separation
C5	−2.521	"I felt that everything I did was an effort."; "I felt hands trembling."; "I enjoyed life."; "I was happy."; "In the last month, how often have you been upset because of something that happened unexpectedly?"
C7	+2.237	"I felt hands trembling."; "In the last month, how often have you been able to control irritations in your life?"; "I could not get going."; "In the last month, how often have you been upset because of something that happened unexpectedly?"; "I felt that everything I did was an effort."
C1	+2.064	"How often do you wake up too early and cannot get back to sleep?"; "How often do you wake up during the night and have trouble getting back to sleep?"; "How often do you have trouble falling asleep?"; "My sleep was restless."; "How often do you feel really rested when you wake up in the morning?"

Case XIN-1

Mahalanobis distance: 6.5592

Empirical rank: 1048/24,292 (top 4.314%)

Non-zero items: 35/37

Dominant component	Z-score	Highest-loading items contributing to separation
C6	+3.402	"Do you have poor appetite or tend to overeat?"; "Do you have little interest or pleasure in doing things?"; "Trouble relaxing"; "In the last month, how often have you felt that you were on top of things?"; "Do you have trouble falling or staying asleep, or do you sleep too much?"
C12	−2.068	"Problems waking up too early"; "Do you feel tired or have little energy?"; "Do you have trouble falling or staying asleep, or do you sleep too much?"; "Have you had thoughts of self-harm or felt that you would be better off dead?"; "Do you have poor appetite or tend to overeat?"
C3	−1.716	"Have you been moving or speaking so slowly that other people have noticed, or the opposite being fidgety or restless?"; "Feeling afraid as if something awful might happen"; "Have you had thoughts of self-harm or felt that you would be better off dead?"; "Do you have trouble concentrating on things, such as reading, work, or watching television?"; "Do you have little interest or pleasure in doing things?"

Analytic summary.

This respondent's outlying position was driven by nonzero responses on low interest/pleasure, trouble relaxing, trouble falling or staying asleep, tiredness/low energy, and the self-harm/better-off-dead item, together with zero responses on appetite disturbance and early waking. Additional contribution came from fear-related, concentration-related, and moving/speaking-slowly or restless items. The unusual feature is the co-occurrence of these present and absent items within the same response pattern.

Case XIN-2

Mahalanobis distance: 7.3665

Empirical rank: 574/24,292 (top 2.363%)

Non-zero items: 28/37

Analytic summary.

This respondent's outlying position was driven by nonzero responses on the moving/speaking-slowly item, tiredness/low energy, low interest/pleasure, appetite-related items, and mild restlessness, together with zero responses on concentration difficulty, fear that something awful might happen,

medRxiv preprint doi: https://doi.org/10.64898/2026.05.22.26353902; this version posted June 12, 2026. The copyright holder for this preprint (which was not certified by peer review) is the author/funder, who has granted medRxiv a license to display the preprint in perpetuity. This article is a US Government work. It is not subject to copyright under 17 USC 105 and is also made available for use under a CC0 license.

Dominant component	Z-score	Highest-loading items contributing to separation
C11	−2.903	"Do you have trouble concentrating on things, such as reading, work, or watching television?"; "Being so restless that it is hard to sit still"; "In the last month, how often have you felt nervous and stressed?"; "In the last month, how often have you felt that you were unable to control the important things in your life?"; "In the last month, how often have you been able to control the way you spend your time?"
C3	+2.450	"Have you been moving or speaking so slowly that other people have noticed, or the opposite being fidgety or restless?"; "Feeling afraid as if something awful might happen"; "Have you had thoughts of self-harm or felt that you would be better off dead?"; "Do you have trouble concentrating on things, such as reading, work, or watching television?"; "Do you have little interest or pleasure in doing things?"
C2	−2.335	"Do you feel down, depressed, or hopeless?"; "Do you feel bad about yourself or that you are a failure or have let yourself or your family down?"; "Do you feel tired or have little energy?"; "Do you have little interest or pleasure in doing things?"; "Do you have poor appetite or tend to overeat?"

and the self-harm/better-off-dead item. The unusual feature is the co-occurrence of these present and absent items within the same response pattern.

Case XIN-3

Mahalanobis distance: 6.6957
Empirical rank: 958/24,292 (top 3.944%)
Non-zero items: 23/37

Dominant component	Z-score	Highest-loading items contributing to separation
C11	+2.274	"Do you have trouble concentrating on things, such as reading, work, or watching television?"; "Being so restless that it is hard to sit still"; "In the last month, how often have you felt nervous and stressed?"; "In the last month, how often have you felt that you were unable to control the important things in your life?"; "In the last month, how often have you been able to control the way you spend your time?"
C9	+2.215	"Do you feel bad about yourself or that you are a failure or have let yourself or your family down?"; "Do you feel tired or have little energy?"; "Have you been moving or speaking so slowly that other people have noticed, or the opposite being fidgety or restless?"; "Do you have poor appetite or tend to overeat?"; "Feeling nervous, anxious or on edge"
C12	−2.085	"Problems waking up too early"; "Do you feel tired or have little energy?"; "Do you have trouble falling or staying asleep, or do you sleep too much?"; "Have you had thoughts of self-harm or felt that you would be better off dead?"; "Do you have poor appetite or tend to overeat?"

Analytic summary.

This respondent's outlying position was driven by nonzero responses on concentration difficulty, nervous/stressed items, sleep difficulty, mild appetite-related items, and mild anxious/on-edge items, together with weak or zero responses on restlessness, self-reproach, tiredness/low energy, moving/speaking-slowly, the self-harm/better-off-dead item, and early waking. The unusual feature is the co-occurrence of these present and absent items within the same response pattern.

Case XIN-4

Mahalanobis distance: 6.3804
Empirical rank: 1197/24,292 (top 4.928%)

medRxiv preprint doi: https://doi.org/10.64898/2026.05.22.26353902; this version posted June 12, 2026. The copyright holder for this preprint (which was not certified by peer review) is the author/funder, who has granted medRxiv a license to display the preprint in perpetuity. This article is a US Government work. It is not subject to copyright under 17 USC 105 and is also made available for use under a CC0 license.

Non-zero items: 29/37

Dominant component	Z-score	Highest-loading items contributing to separation
C3	−2.911	"Have you been moving or speaking so slowly that other people have noticed, or the opposite being fidgety or restless?"; "Feeling afraid as if something awful might happen"; "Have you had thoughts of self-harm or felt that you would be better off dead?"; "Do you have trouble concentrating on things, such as reading, work, or watching television?"; "Do you have little interest or pleasure in doing things?"
C1	+1.506	"How satisfied are you with your current sleep pattern?"; "To what extent do you consider your sleep problem to interfere with your daily functioning?"; "How noticeable to others do you think your sleep problem is in terms of impairing the quality of your life?"; "Feeling afraid as if something awful might happen"; "Becoming easily annoyed or irritable"
C13	−1.460	"Being so restless that it is hard to sit still"; "Worrying too much about different things"; "In the last month, how often have you dealt successfully with day-to-day problems and annoyances?"; "Do you feel bad about yourself or that you are a failure or have let yourself or your family down?"; "In the last month, how often have you felt that you were unable to control the important things in your life?"

Analytic summary.

This respondent's outlying position was driven by endorsement of the self-harm/better-off-dead item together with subjective sleep dissatisfaction, sleep interference, and noticeability of the sleep problem, while moving/speaking-slowly, fear-that-something-awful-might-happen, concentration difficulty, restlessness, and worrying-too-much items remained weak or absent. The unusual feature is the co-occurrence of the self-harm/better-off-dead item with selected sleep-related items and weak responses on several other item groups.

Case XIN-5

Mahalanobis distance: 6.3590

Empirical rank: 1214/24,292 (top 4.998%)

Non-zero items: 22/37

Dominant component	Z-score	Highest-loading items contributing to separation
C5	−2.071	"Worrying too much about different things"; "How noticeable to others do you think your sleep problem is in terms of impairing the quality of your life?"; "Do you have trouble concentrating on things, such as reading, work, or watching television?"; "Difficulty falling asleep"; "Difficulty staying asleep"
C6	+1.992	"Do you have poor appetite or tend to overeat?"; "Do you have little interest or pleasure in doing things?"; "Trouble relaxing"; "In the last month, how often have you felt that you were on top of things?"; "Do you have trouble falling or staying asleep, or do you sleep too much?"
C1	+1.900	"How satisfied are you with your current sleep pattern?"; "To what extent do you consider your sleep problem to interfere with your daily functioning?"; "How noticeable to others do you think your sleep problem is in terms of impairing the quality of your life?"; "Feeling afraid as if something awful might happen"; "Becoming easily annoyed or irritable"

Analytic summary.

This respondent's outlying position was driven by nonzero responses on worrying-too-much items, sleep-problem noticeability, sleep-related interference, and mild low-interest/pleasure items, to-

medRxiv preprint doi: https://doi.org/10.64898/2026.05.22.26353902; this version posted June 12, 2026. The copyright holder for this preprint (which was not certified by peer review) is the author/funder, who has granted medRxiv a license to display the preprint in perpetuity. This article is a US Government work. It is not subject to copyright under 17 USC 105 and is also made available for use under a CC0 license.

Spitzer RL, Kroenke K, Williams JBW, Löwe B. A Brief Measure for Assessing Generalized Anxiety Disorder. Archives of Internal Medicine. 2006; 166:1092. doi: 10.1001/archinte.166.10.1092.

Steffick DE. Documentation of affective functioning measures in the Health and Retirement Study (HRS/AHEAD Documentation Report DR-005). Survey Research Center, University of Michigan; 2000.

Su Z, Liu R, Wei Y, Zhang R, Xu X, Wang Y, Zhu Y, Wang L, Liang L, Wang F, Zhang X. Temporal dynamics in psychological assessments: a novel dataset with scales and response times. Scientific Data. 2024 Sep; 11(1):1046. doi: 10.1038/s41597-024-03888-8.

Watson D. Rethinking the mood and anxiety disorders: A quantitative hierarchical model for DSM-V. Journal of Abnormal Psychology. 2005; 114(4):522–536. doi: 10.1037/0021-843X.114.4.522.

Xiao S, Liu Z, Zhang P, Mu N. C-Pack: Packaged Resources To Advance General Chinese Embedding. arXiv preprint arXiv:230907597. 2023; .

Supplementary Appendix 1. Structured analytic summaries of retained multivariate outliers

Scope and interpretive constraints

The case summaries below are analytic descriptions of response configurations in the finalized semantically aligned subspace. They are not clinical case formulations, diagnostic judgments, or treatment recommendations. Cases were retained only if they exceeded the cohort-specific empirical Mahalanobis threshold after Ledoit-Wolf covariance regularization *and* did not endorse any item at the Likert ceiling on any instrument. Age and sex were regressed out before subspace scoring. Respondent study identifiers have been removed in accordance with data use requirements. Cases are identified by their anonymized case labels only.

Because several questionnaire items were reverse-coded during preprocessing, interpretation is based on the joint configuration of items and component structure, rather than on any single coded response in isolation. For clarity, item labels are reported in their original questionnaire wording.

Older-adult cohort: HRS

Cohort size: 4,413

Retained subspace dimensionality: 10

Retained cross-instrument outliers: 7

Case HRS-1

Mahalanobis distance: 5.2901

Empirical rank: 84/4,413 (top 1.903%)

Non-zero items: 22/27

Dominant component	Z-score	Highest-loading items contributing to separation
C5	−3.202	“I felt that everything I did was an effort.”; “I felt hands trembling.”; “I enjoyed life.”; “I was happy.”; “In the last month, how often have you been upset because of something that happened unexpectedly?”
C3	−1.819	“I had a fear of the worst happening.”; “I felt lonely.”; “I had a fear of dying.”; “I felt sad.”; “I felt depressed.”
C9	−1.687	“How often do you feel really rested when you wake up in the morning?”; “In the last month, how often have you felt that things were going your way?”; “In the last month, how often have you found that you could not cope with all the things that you had to do?”; “How often do you have trouble falling asleep?”; “In the last month, how often have you felt nervous and stressed?”

medRxiv preprint doi: https://doi.org/10.64898/2026.05.22.26353902; this version posted June 12, 2026. The copyright holder for this preprint (which was not certified by peer review) is the author/funder, who has granted medRxiv a license to display the preprint in perpetuity. This article is a US Government work. It is not subject to copyright under 17 USC 105 and is also made available for use under a CC0 license.

Analytic summary.

This respondent's outlying position was driven by nonzero responses on upset-over-unexpected-events, fear-of-the-worst-happening, fear-of-dying, hands-trembling, trouble-falling-asleep, and nervous/stressed items, together with zero responses on loneliness-, sadness-, depression-, and effort-labeled items. The positive-valence items "I enjoyed life" and "I was happy," as well as restfulness and coping-related items, also contributed to the separation. The unusual feature is the co-occurrence of these present and absent items within the same response pattern.

Case HRS-2

Mahalanobis distance: 5.1196

Empirical rank: 128/4,413 (top 2.901%)

Non-zero items: 21/27

Dominant component	Z-score	Highest-loading items contributing to separation
C2	+2.690	"I enjoyed life."; "I was happy."; "How often do you feel really rested when you wake up in the morning?"; "I felt that everything I did was an effort."; "In the last month, how often have you felt that you were unable to control the important things in your life?"
C9	−2.220	"How often do you feel really rested when you wake up in the morning?"; "In the last month, how often have you felt that things were going your way?"; "In the last month, how often have you found that you could not cope with all the things that you had to do?"; "How often do you have trouble falling asleep?"; "In the last month, how often have you felt nervous and stressed?"
C5	−2.195	"I felt that everything I did was an effort."; "I felt hands trembling."; "I enjoyed life."; "I was happy."; "In the last month, how often have you been upset because of something that happened unexpectedly?"

Analytic summary.

This respondent's outlying position was driven by nonzero responses on the positive-valence items "I enjoyed life" and "I was happy," restfulness-on-waking, trouble-falling-asleep, things-going-your-way, upset-over-unexpected-events, and could-not-cope items, together with zero responses on the effort item. Hands-trembling also contributed at lower level. The unusual feature is the joint placement of positive-valence, sleep-related, and stress-related items together with a zero response on the effort item.

Case HRS-3

Mahalanobis distance: 5.2662

Empirical rank: 95/4,413 (top 2.153%)

Non-zero items: 22/27

Analytic summary.

This respondent's outlying position was driven by nonzero responses on fear-of-the-worst-happening, fear-of-dying, loneliness-, sadness-, and depression-labeled items, upset-over-unexpected-events, trouble-falling-asleep, and control-related items, together with zero responses on "I enjoyed life," "I was happy," and the effort item. Restfulness-on-waking also contributed. The unusual feature is the co-occurrence of nonzero responses on several fear-, sadness-, and control-related items with zero responses on the positive-valence and effort items.

Case HRS-4

Mahalanobis distance: 4.9443

Empirical rank: 191/4,413 (top 4.328%)

medRxiv preprint doi: https://doi.org/10.64898/2026.05.22.26353902; this version posted June 12, 2026. The copyright holder for this preprint (which was not certified by peer review) is the author/funder, who has granted medRxiv a license to display the preprint in perpetuity. This article is a US Government work. It is not subject to copyright under 17 USC 105 and is also made available for use under a CC0 license.

Dominant component	Z-score	Highest-loading items contributing to separation
C2	−2.915	"I enjoyed life."; "I was happy."; "How often do you feel really rested when you wake up in the morning?"; "I felt that everything I did was an effort."; "In the last month, how often have you felt that you were unable to control the important things in your life?"
C3	+2.478	"I had a fear of the worst happening."; "I felt lonely."; "I had a fear of dying."; "I felt sad."; "I felt depressed."
C6	+1.948	"In the last month, how often have you been upset because of something that happened unexpectedly?"; "In the last month, how often have you felt confident about your ability to handle your personal problems?"; "I had a fear of dying."; "How often do you have trouble falling asleep?"; "In the last month, how often have you been able to control irritations in your life?"

Non-zero items: 22/27

Dominant component	Z-score	Highest-loading items contributing to separation
C2	−1.847	"I enjoyed life."; "I was happy."; "How often do you feel really rested when you wake up in the morning?"; "I felt that everything I did was an effort."; "In the last month, how often have you felt that you were unable to control the important things in your life?"
C3	+1.619	"I had a fear of the worst happening."; "I felt lonely."; "I had a fear of dying."; "I felt sad."; "I felt depressed."
C0	+1.607	"I felt sad."; "I felt lonely."; "I felt faint."; "I was happy."; "I felt depressed."

Analytic summary.

This respondent's outlying position was driven by nonzero responses on fear-of-the-worst-happening, loneliness-, depression-, and faintness-labeled items, together with zero responses on "I enjoyed life," "I was happy," the effort item, and the sadness item in one dominant component. Restfulness-on-waking and inability-to-control-important-things also contributed. The unusual feature is the joint placement of faintness-, fear-, and selected mood-labeled items with zero responses on positive-valence and effort items.

Case HRS-5

Mahalanobis distance: 5.1654
Empirical rank: 120/4,413 (top 2.719%)
Non-zero items: 22/27

Dominant component	Z-score	Highest-loading items contributing to separation
C2	−2.903	"I enjoyed life."; "I was happy."; "How often do you feel really rested when you wake up in the morning?"; "I felt that everything I did was an effort."; "In the last month, how often have you felt that you were unable to control the important things in your life?"
C3	+2.893	"I had a fear of the worst happening."; "I felt lonely."; "I had a fear of dying."; "I felt sad."; "I felt depressed."
C6	+2.581	"In the last month, how often have you been upset because of something that happened unexpectedly?"; "In the last month, how often have you felt confident about your ability to handle your personal problems?"; "I had a fear of dying."; "How often do you have trouble falling asleep?"; "In the last month, how often have you been able to control irritations in your life?"

medRxiv preprint doi: https://doi.org/10.64898/2026.05.22.26353902; this version posted June 12, 2026. The copyright holder for this preprint (which was not certified by peer review) is the author/funder, who has granted medRxiv a license to display the preprint in perpetuity. This article is a US Government work. It is not subject to copyright under 17 USC 105 and is also made available for use under a CC0 license.

Analytic summary.

This respondent's outlying position was driven by nonzero responses on loneliness-, sadness-, and depression-labeled items, fear-of-dying, upset-over-unexpected-events, confidence/control-related items, and trouble-falling-asleep, together with zero responses on "I enjoyed life," "I was happy," and the effort item. Restfulness-on-waking also contributed. The unusual feature is the co-occurrence of nonzero responses on several sadness-, fear-, and control-related items with zero responses on the positive-valence and effort items.

Case HRS-6

Mahalanobis distance: 5.0286

Empirical rank: 156/4,413 (top 3.535%)

Non-zero items: 22/27

Dominant component	Z-score	Highest-loading items contributing to separation
C6	−3.325	"In the last month, how often have you been upset because of something that happened unexpectedly?"; "In the last month, how often have you felt confident about your ability to handle your personal problems?"; "I had a fear of dying."; "How often do you have trouble falling asleep?"; "In the last month, how often have you been able to control irritations in your life?"
C4	+2.877	"I could not get going."; "In the last month, how often have you found that you could not cope with all the things that you had to do?"; "In the last month, how often have you felt nervous and stressed?"; "I had a fear of the worst happening."; "In the last month, how often have you felt difficulties were piling up so high that you could not overcome them?"
C3	−2.107	"I had a fear of the worst happening."; "I felt lonely."; "I had a fear of dying."; "I felt sad."; "I felt depressed."

Analytic summary.

This respondent's outlying position was driven by nonzero responses on upset-over-unexpected-events, inability-to-cope, difficulties-piling-up, nervous/stressed, "I could not get going," and fear-related items, together with zero responses on loneliness-, sadness-, and depression-labeled items. Trouble-falling-asleep and control-related items also contributed. The unusual feature is the co-occurrence of nonzero responses on several stress-, coping-, and fear-related items with zero responses on several mood-labeled items.

Case HRS-7

Mahalanobis distance: 5.2535

Empirical rank: 99/4,413 (top 2.243%)

Non-zero items: 22/27

Analytic summary.

This respondent's outlying position was driven by nonzero responses on hands-trembling, upset-over-unexpected-events, early-waking, night-waking, trouble-falling-asleep, restless-sleep, and restfulness-on-waking items, together with zero responses on "I could not get going" and the effort item in two dominant components. The positive-valence items "I enjoyed life" and "I was happy" also contributed. The unusual feature is the joint placement of trembling-, stress-, positive-valence-, and multiple sleep-related items together with zero responses on selected activation-related items.

Younger-adult cohort: Xinxiang

Cohort size: 24,292

Retained subspace dimensionality: 16

Retained cross-instrument outliers: 7

medRxiv preprint doi: https://doi.org/10.64898/2026.05.22.26353902; this version posted June 12, 2026. The copyright holder for this preprint (which was not certified by peer review) is the author/funder, who has granted medRxiv a license to display the preprint in perpetuity. This article is a US Government work. It is not subject to copyright under 17 USC 105 and is also made available for use under a CC0 license.

Dominant component	Z-score	Highest-loading items contributing to separation
C5	−2.521	"I felt that everything I did was an effort."; "I felt hands trembling."; "I enjoyed life."; "I was happy."; "In the last month, how often have you been upset because of something that happened unexpectedly?"
C7	+2.237	"I felt hands trembling."; "In the last month, how often have you been able to control irritations in your life?"; "I could not get going."; "In the last month, how often have you been upset because of something that happened unexpectedly?"; "I felt that everything I did was an effort."
C1	+2.064	"How often do you wake up too early and cannot get back to sleep?"; "How often do you wake up during the night and have trouble getting back to sleep?"; "How often do you have trouble falling asleep?"; "My sleep was restless."; "How often do you feel really rested when you wake up in the morning?"

Case XIN-1

Mahalanobis distance: 6.5592

Empirical rank: 1048/24,292 (top 4.314%)

Non-zero items: 35/37

Dominant component	Z-score	Highest-loading items contributing to separation
C6	+3.402	"Do you have poor appetite or tend to overeat?"; "Do you have little interest or pleasure in doing things?"; "Trouble relaxing"; "In the last month, how often have you felt that you were on top of things?"; "Do you have trouble falling or staying asleep, or do you sleep too much?"
C12	−2.068	"Problems waking up too early"; "Do you feel tired or have little energy?"; "Do you have trouble falling or staying asleep, or do you sleep too much?"; "Have you had thoughts of self-harm or felt that you would be better off dead?"; "Do you have poor appetite or tend to overeat?"
C3	−1.716	"Have you been moving or speaking so slowly that other people have noticed, or the opposite being fidgety or restless?"; "Feeling afraid as if something awful might happen"; "Have you had thoughts of self-harm or felt that you would be better off dead?"; "Do you have trouble concentrating on things, such as reading, work, or watching television?"; "Do you have little interest or pleasure in doing things?"

Analytic summary.

This respondent's outlying position was driven by nonzero responses on low interest/pleasure, trouble relaxing, trouble falling or staying asleep, tiredness/low energy, and the self-harm/better-off-dead item, together with zero responses on appetite disturbance and early waking. Additional contribution came from fear-related, concentration-related, and moving/speaking-slowly or restless items. The unusual feature is the co-occurrence of these present and absent items within the same response pattern.

Case XIN-2

Mahalanobis distance: 7.3665

Empirical rank: 574/24,292 (top 2.363%)

Non-zero items: 28/37

Analytic summary.

This respondent's outlying position was driven by nonzero responses on the moving/speaking-slowly item, tiredness/low energy, low interest/pleasure, appetite-related items, and mild restlessness, together with zero responses on concentration difficulty, fear that something awful might happen,

medRxiv preprint doi: https://doi.org/10.64898/2026.05.22.26353902; this version posted June 12, 2026. The copyright holder for this preprint (which was not certified by peer review) is the author/funder, who has granted medRxiv a license to display the preprint in perpetuity. This article is a US Government work. It is not subject to copyright under 17 USC 105 and is also made available for use under a CC0 license.

Dominant component	Z-score	Highest-loading items contributing to separation
C11	−2.903	"Do you have trouble concentrating on things, such as reading, work, or watching television?"; "Being so restless that it is hard to sit still"; "In the last month, how often have you felt nervous and stressed?"; "In the last month, how often have you felt that you were unable to control the important things in your life?"; "In the last month, how often have you been able to control the way you spend your time?"
C3	+2.450	"Have you been moving or speaking so slowly that other people have noticed, or the opposite being fidgety or restless?"; "Feeling afraid as if something awful might happen"; "Have you had thoughts of self-harm or felt that you would be better off dead?"; "Do you have trouble concentrating on things, such as reading, work, or watching television?"; "Do you have little interest or pleasure in doing things?"
C2	−2.335	"Do you feel down, depressed, or hopeless?"; "Do you feel bad about yourself or that you are a failure or have let yourself or your family down?"; "Do you feel tired or have little energy?"; "Do you have little interest or pleasure in doing things?"; "Do you have poor appetite or tend to overeat?"

595 and the self-harm/better-off-dead item. The unusual feature is the co-occurrence of these present and absent items within the same response pattern.

Case XIN-3

Mahalanobis distance: 6.6957
Empirical rank: 958/24,292 (top 3.944%)
Non-zero items: 23/37

Dominant component	Z-score	Highest-loading items contributing to separation
C11	+2.274	"Do you have trouble concentrating on things, such as reading, work, or watching television?"; "Being so restless that it is hard to sit still"; "In the last month, how often have you felt nervous and stressed?"; "In the last month, how often have you felt that you were unable to control the important things in your life?"; "In the last month, how often have you been able to control the way you spend your time?"
C9	+2.215	"Do you feel bad about yourself or that you are a failure or have let yourself or your family down?"; "Do you feel tired or have little energy?"; "Have you been moving or speaking so slowly that other people have noticed, or the opposite being fidgety or restless?"; "Do you have poor appetite or tend to overeat?"; "Feeling nervous, anxious or on edge"
C12	−2.085	"Problems waking up too early"; "Do you feel tired or have little energy?"; "Do you have trouble falling or staying asleep, or do you sleep too much?"; "Have you had thoughts of self-harm or felt that you would be better off dead?"; "Do you have poor appetite or tend to overeat?"

Analytic summary.

This respondent's outlying position was driven by nonzero responses on concentration difficulty, nervous/stressed items, sleep difficulty, mild appetite-related items, and mild anxious/on-edge items, together with weak or zero responses on restlessness, self-reproach, tiredness/low energy, moving/speaking-slowly, the self-harm/better-off-dead item, and early waking. The unusual feature is the co-occurrence of these present and absent items within the same response pattern.

Case XIN-4

Mahalanobis distance: 6.3804
Empirical rank: 1197/24,292 (top 4.928%)

medRxiv preprint doi: https://doi.org/10.64898/2026.05.22.26353902; this version posted June 12, 2026. The copyright holder for this preprint (which was not certified by peer review) is the author/funder, who has granted medRxiv a license to display the preprint in perpetuity. This article is a US Government work. It is not subject to copyright under 17 USC 105 and is also made available for use under a CC0 license.

Non-zero items: 29/37

Dominant component	Z-score	Highest-loading items contributing to separation
C3	−2.911	"Have you been moving or speaking so slowly that other people have noticed, or the opposite being fidgety or restless?"; "Feeling afraid as if something awful might happen"; "Have you had thoughts of self-harm or felt that you would be better off dead?"; "Do you have trouble concentrating on things, such as reading, work, or watching television?"; "Do you have little interest or pleasure in doing things?"
C1	+1.506	"How satisfied are you with your current sleep pattern?"; "To what extent do you consider your sleep problem to interfere with your daily functioning?"; "How noticeable to others do you think your sleep problem is in terms of impairing the quality of your life?"; "Feeling afraid as if something awful might happen"; "Becoming easily annoyed or irritable"
C13	−1.460	"Being so restless that it is hard to sit still"; "Worrying too much about different things"; "In the last month, how often have you dealt successfully with day-to-day problems and annoyances?"; "Do you feel bad about yourself or that you are a failure or have let yourself or your family down?"; "In the last month, how often have you felt that you were unable to control the important things in your life?"

Analytic summary.

This respondent's outlying position was driven by endorsement of the self-harm/better-off-dead item together with subjective sleep dissatisfaction, sleep interference, and noticeability of the sleep problem, while moving/speaking-slowly, fear-that-something-awful-might-happen, concentration difficulty, restlessness, and worrying-too-much items remained weak or absent. The unusual feature is the co-occurrence of the self-harm/better-off-dead item with selected sleep-related items and weak responses on several other item groups.

Case XIN-5

Mahalanobis distance: 6.3590
Empirical rank: 1214/24,292 (top 4.998%)
Non-zero items: 22/37

Dominant component	Z-score	Highest-loading items contributing to separation
C5	−2.071	"Worrying too much about different things"; "How noticeable to others do you think your sleep problem is in terms of impairing the quality of your life?"; "Do you have trouble concentrating on things, such as reading, work, or watching television?"; "Difficulty falling asleep"; "Difficulty staying asleep"
C6	+1.992	"Do you have poor appetite or tend to overeat?"; "Do you have little interest or pleasure in doing things?"; "Trouble relaxing"; "In the last month, how often have you felt that you were on top of things?"; "Do you have trouble falling or staying asleep, or do you sleep too much?"
C1	+1.900	"How satisfied are you with your current sleep pattern?"; "To what extent do you consider your sleep problem to interfere with your daily functioning?"; "How noticeable to others do you think your sleep problem is in terms of impairing the quality of your life?"; "Feeling afraid as if something awful might happen"; "Becoming easily annoyed or irritable"

Analytic summary.

This respondent's outlying position was driven by nonzero responses on worrying-too-much items, sleep-problem noticeability, sleep-related interference, and mild low-interest/pleasure items, to-

medRxiv preprint doi: https://doi.org/10.64898/2026.05.22.26353902; this version posted June 12, 2026. The copyright holder for this preprint (which was not certified by peer review) is the author/funder, who has granted medRxiv a license to display the preprint in perpetuity. This article is a US Government work. It is not subject to copyright under 17 USC 105 and is also made available for use under a CC0 license.

gether with weak or zero responses on difficulty falling asleep, difficulty staying asleep, trouble relaxing, fear-that-something-awful-might-happen, and irritability. The unusual feature is the co-occurrence of these present and absent items within the same response pattern.

Case XIN-6

Mahalanobis distance: 7.2424
Empirical rank: 629/24,292 (top 2.589%)
Non-zero items: 24/37

Dominant component	Z-score	Highest-loading items contributing to separation
C2	−3.837	"Do you feel down, depressed, or hopeless?"; "Do you feel bad about yourself or that you are a failure or have let yourself or your family down?"; "Do you feel tired or have little energy?"; "Do you have little interest or pleasure in doing things?"; "Do you have poor appetite or tend to overeat?"
C7	−2.676	"Do you have poor appetite or tend to overeat?"; "Becoming easily annoyed or irritable"; "In the last month, how often have you been upset because of something that happened unexpectedly?"; "Do you have trouble concentrating on things, such as reading, work, or watching television?"; "Have you had thoughts of self-harm or felt that you would be better off dead?"
C12	−2.392	"Problems waking up too early"; "Do you feel tired or have little energy?"; "Do you have trouble falling or staying asleep, or do you sleep too much?"; "Have you had thoughts of self-harm or felt that you would be better off dead?"; "Do you have poor appetite or tend to overeat?"

Analytic summary.

This respondent's outlying position was driven by nonzero responses on depressed/hopeless, self-reproach, tiredness/low energy, low interest/pleasure, appetite-related items, upset over unexpected events, trouble sleeping, and the self-harm/better-off-dead item, together with weak or zero responses on irritability, concentration difficulty, and early waking. This respondent also exhibited the most extreme single-component deviation among the retained Xinxiang cases ($Z = -3.837$ on Component 2), which is why it is highlighted in the heatmap. The unusual feature is the co-occurrence of these present and absent items within the same response pattern.

Case XIN-7

Mahalanobis distance: 6.3878
Empirical rank: 1192/24,292 (top 4.907%)
Non-zero items: 29/37

Analytic summary.

This respondent's outlying position was driven by nonzero responses on subjective sleep dissatisfaction, sleep interference, sleep-problem noticeability, and difficulty falling asleep, with smaller contributions from restlessness, worrying-too-much items, and self-reproach. In contrast, fear-that-something-awful-might-happen, difficulty staying asleep, and early waking were weak or absent. The unusual feature is the co-occurrence of these present and absent items within the same response pattern.

Cross-case summary

Across the retained cases, the dominant signal was not uniformly high symptom severity but unusual cross-domain response configurations. In HRS, several respondents combined nonzero fear-related, trembling/faintness-related, and sleep-related items with weak or zero responses on sadness-, loneliness-, and depression-labeled items. In Xinxiang, several respondents combined

medRxiv preprint doi: https://doi.org/10.64898/2026.05.22.26353902; this version posted June 12, 2026. The copyright holder for this preprint (which was not certified by peer review) is the author/funder, who has granted medRxiv a license to display the preprint in perpetuity. This article is a US Government work. It is not subject to copyright under 17 USC 105 and is also made available for use under a CC0 license.

Dominant component	Z-score	Highest-loading items contributing to separation
C1	+3.698	"How satisfied are you with your current sleep pattern?"; "To what extent do you consider your sleep problem to interfere with your daily functioning?"; "How noticeable to others do you think your sleep problem is in terms of impairing the quality of your life?"; "Feeling afraid as if something awful might happen"; "Becoming easily annoyed or irritable"
C13	+2.200	"Being so restless that it is hard to sit still"; "Worrying too much about different things"; "In the last month, how often have you dealt successfully with day-to-day problems and annoyances?"; "Do you feel bad about yourself or that you are a failure or have let yourself or your family down?"; "In the last month, how often have you felt that you were unable to control the important things in your life?"
C0	+1.533	"Difficulty falling asleep"; "Difficulty staying asleep"; "In the last month, how often have you felt that you were effectively coping with important changes that were occurring in your life?"; "In the last month, how often have you felt that things were going your way?"; "Problems waking up too early"

selected mood-, sleep-, stress-, concentration-, movement/restlessness-, appetite-, or self-harm-related items without the broader co-occurrence pattern that would be more strongly prioritized by additive screening. In all cases the anomaly is geometric—a position unusual in the joint covariance space—rather than scalar.
